# Comparison of COPD primary care in England, Scotland, Wales, and Northern Ireland

**DOI:** 10.1038/s41533-022-00305-8

**Published:** 2022-10-25

**Authors:** Philip W. Stone, Katherine Hickman, Steve Holmes, Johanna R. Feary, Jennifer K. Quint

**Affiliations:** 1 National Heart and Lung Institute, Imperial College London, London, UK; 2 West Yorkshire & Harrogate Health and Care Partnership, Bradford, UK; 3 The Park Medical Practice, Shepton Mallet, UK

**Keywords:** Chronic obstructive pulmonary disease, Health services

## Abstract

Currently the National Asthma and COPD audit programme (NACAP) only undertakes audit of COPD primary care in Wales due to its near complete data coverage. We aimed to determine if the quality of COPD primary care in the other UK nations is comparable with Wales. We found that English, Scottish, and Northern Irish practices were significantly worse than Welsh practices at recording coded lung function parameters used in COPD diagnosis (ORs: 0.51 [0.43–0.59], 0.29 [0.23–0.36], 0.42 [0.31–0.58], respectively) and referring appropriate patients for pulmonary rehabilitation (ORs: 0.10 [0.09–0.11], 0.12 [0.11–0.14], 0.22 [0.19–0.25], respectively). Completing national audits of primary care in Wales only may have led to improvements in care, or at least improvements in the recording of care in Wales that are not occurring elsewhere in the UK. This highlights the potential importance of audit in improving care quality and accurate recording of that care.

## Introduction

Chronic Obstructive Pulmonary Disease (COPD) is a disease characterised by respiratory symptoms such as breathlessness, cough, and sputum production, as well as airflow limitation due to damage to the airway and/or alveoli^1,2^. COPD is the second most common lung disease in the United Kingdom (UK) with approximately 1.2 million people diagnosed^3^, leading to an estimated annual healthcare cost of £1.8 billion^4^. COPD is the 4th and 5th most common cause of death for men and women, respectively^5^, and the UK has the 3rd highest mortality rate for COPD in Europe, and the 12th highest mortality rate in the world^3^.

Healthcare is a devolved matter in the UK, with each of the four constituent countries being responsible for healthcare within their borders. While per capita healthcare spending is similar in each of the four UK nations^6^, healthcare commissioning and incentivisation can differ between them, which may lead to differences in peformance^7^.

The estimated prevalence of COPD is similar between the UK countries, with England, Northern Ireland, and Wales having estimates of 2.0%, 2.1%, and 2.2%, respectively; however, Scotland does have a slightly higher estimated prevalence of 2.4%^3^. COPD mortality is similar between England, Wales, and Northern Ireland, but Scotland has higher than expected mortality for COPD^3^. Scottish women and men with COPD have 32% and 12%, respectively, higher mortality than would be expected based on age-standardised mortality ratios for the UK^3^.

In 2017, the National Asthma and COPD Audit Programme (NACAP) conducted an audit of primary care comprising 94% of all primary care practices in Wales^8^. While it had been desired to include all UK countries in the audit, patient confidentiality concerns arising from the ‘care.data’ project have resulted in a block to the sharing of patient data from English practices, and the proportion of practices from Scotland and Northern Ireland agreeing to participate was too low to ensure generalisability of results. Therefore Wales is the only country to have received national audits of COPD primary care so far^9^. As a result of being the only participant in the primary care audits, there may be an increased awareness of best practice COPD care among Welsh GPs that is not present in the rest of the UK. This in turn may have led to improvements in care in Wales that are not occurring in the rest of the UK.

Therefore, we aimed to determine if Wales is comparable to the other UK nations in terms of COPD primary care by replicating the NACAP 2017 primary care audit in each of the four UK countries using a large UK primary care research database.

## Results

**Fig. 1:**
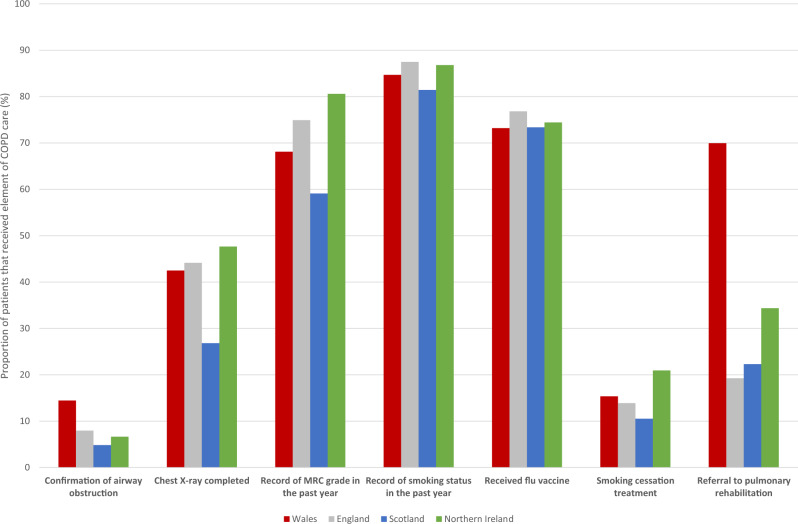
Proportion of patients receiving key items of COPD care in Welsh, English, Scottish, and Northern Irish practices in CPRD GOLD. COPD chronic obstructive pulmonary disease, MRC Medical Research Council.

In the 69 Welsh, 141 English, 74 Scottish, and 21 Northern Irish CPRD GOLD GP practices (15%, 2%, 8%, and 6% of practices in each country, respectively) there were, respectively, 13,587 Welsh, 25,689 English, 13,717 Scottish, and 3771 Northern Irish patients with a diagnosis of COPD (56,764 total patients). A lower proportion of Scottish patients received a chest X-ray in the 6 months prior to or following diagnosis than Welsh patients (26.8% in Scotland vs. 42.5% in Wales), and a substantially greater proportion of Welsh patients were referred for pulmonary rehabilitation than patients from the other countries (70.0% in Wales vs. 19.0%, 22.3%, and 34.4% in England, Scotland, and Northern Ireland, respectively) (Fig. 1).

**Fig. 2:**
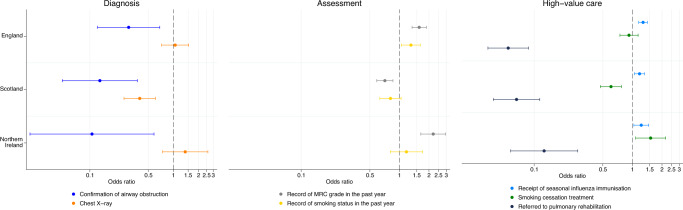
Fully adjusted odds ratios and 95% confidence intervals for receipt of item of COPD care (split into diagnosis, assessment, and high-value care) for each UK country relative to Wales. MRC Medical Research Council.

Relative to Welsh patients, English, Scottish, and Northern Irish COPD patients were significantly less likely to have coded confirmation of airway obstruction (Fig. 2/Table 1). Scottish patients were significantly less likely to have a chest X-ray, but there was no significant difference for English or Northern Irish patients. Scottish patients were significantly less likely to have a record of MRC grade or smoking status in the last year, but English and Northern Irish patients were significantly more likely to have a record of MRC grade and smoking status. English, Scottish, and Northern Irish COPD patients were significantly more likely to have the seasonal influenza vaccine. English and Scottish patients were significantly less likely to receive smoking cessation treatment (referral for a behavioural change intervention and prescription of a stop-smoking drug), whereas Northern Irish patients were significantly more likely to receive it. English, Scottish, and Northern Irish COPD patients were substantially less likely than Welsh patients to receive a referral for pulmonary rehabilitation.

**Table 1:** Odds ratios and 95% confidence intervals for receipt of item of COPD care for each UK country relative to Wales in crude and adjusted models.

	Odds ratio (95% confidence interval)		
	Crude	Age and gender adjusted	Age, gender, and comorbidities adjusted
**Confirmation of airway obstruction**^a^			
Wales	1	1	1
England	0.30 (0.13–0.71)	0.30 (0.13–0.71)	**0.29 (0.12–0.69)**
Scotland	0.14 (0.05–0.38)	0.14 (0.05–0.38)	**0.13 (0.05–0.37)**
Northern Ireland	0.11 (0.02–0.59)	0.11 (0.02–0.58)	**0.11 (0.02–0.59)**
**Chest X-ray**^b^			
Wales	1	1	1
England	1.04 (0.72–1.51)	1.05 (0.72–1.52)	1.05 (0.72–1.52)
Scotland	0.41 (0.26–0.63)	0.41 (0.26–0.63)	**0.40 (0.26–0.61)**
Northern Ireland	1.45 (0.78–2.70)	1.45 (0.78–2.69)	1.39 (0.74–2.59)
**Record of MRC grade in the past year**			
Wales	1	1	1
England	1.56 (1.32–1.85)	1.59 (1.34–1.88)	**1.59 (1.34–1.88)**
Scotland	0.71 (0.59–0.86)	0.73 (0.60–0.88)	**0.71 (0.59–0.86)**
Northern Ireland	2.15 (1.61–2.88)	2.23 (1.67–2.99)	**2.21 (1.65–2.95)**
**Record of smoking status in the past year**			
Wales	1	1	1
England	1.27 (1.02–1.58)	1.29 (1.04–1.61)	**1.31 (1.05–1.63)**
Scotland	0.79 (0.62–1.01)	0.78 (0.61–1.00)	0.81 (0.63–1.04)
Northern Ireland	1.18 (0.81–1.71)	1.15 (0.79–1.67)	1.18 (0.81–1.72)
**Receipt of the seasonal influenza immunisation in the last year**			
Wales	1	1	1
England	1.23 (1.11–1.36)	1.25 (1.13–1.39)	**1.28 (1.16–1.42)**
Scotland	1.04 (0.93–1.17)	1.11 (0.99–1.25)	**1.18 (1.05–1.33)**
Northern Ireland	1.11 (0.93–1.32)	1.23 (1.03–1.46)	**1.23 (1.03–1.47)**
**Smoking cessation treatment**^c^			
Wales	1	1	1
England	0.90 (0.73–1.11)	0.91 (0.74–1.13)	0.92 (0.75–1.14)
Scotland	0.62 (0.49–0.79)	0.61 (0.48–0.78)	**0.60 (0.47–0.77)**
Northern Ireland	1.67 (1.18–2.35)	1.61 (1.14–2.28)	**1.53 (1.08–2.17)**
**Referred to pulmonary rehabilitation**^d^			
Wales	1	1	1
England	0.05 (0.03–0.09)	0.05 (0.03–0.09)	**0.05 (0.03–0.09)**
Scotland	0.07 (0.04–0.11)	0.07 (0.04–0.11)	**0.07 (0.04–0.11)**
Northern Ireland	0.13 (0.06–0.29)	0.13 (0.06–0.28)	**0.13 (0.06–0.28)**

^a^Confirmation of airway obstruction defined as record of post-bronchodilator FEV_1_/FVC < 0.7.

^b^Chest X-ray confirmation of diagnosis defined as record of a chest X-ray 6 months prior to or after COPD diagnosis.

^c^Both a behavioural change intervention and a stop-smoking drug.

^d^“Offered” pulmonary rehabilitation counted as a referral, however any patients that declined the offer were not counted as referred.

Bold text inidicate statistically significant values in the final age, gener, and comorbidites adjusted model.

**Fig. 3:**
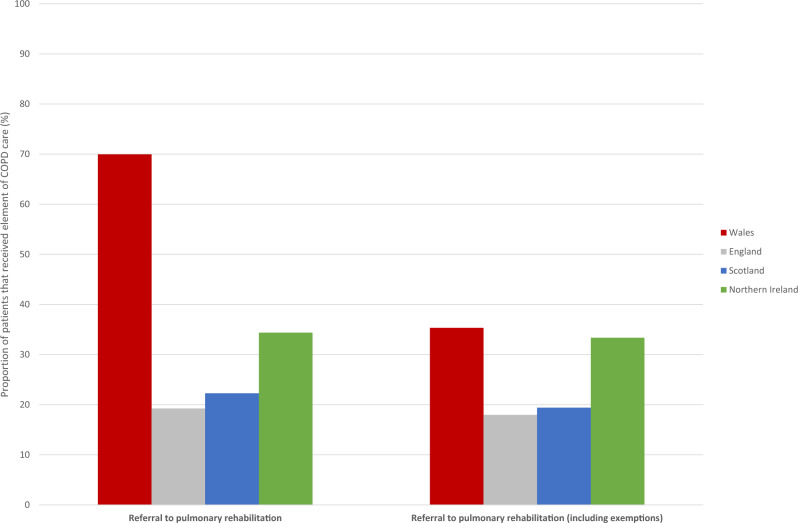
Proportion of patients in Welsh, English, Scottish, and Northern Irish practices referred to pulmonary rehabilitation, excluding exempted patients from the denominator, and including exempted patients in the denominator. COPD chronic obstructive pulmonary disease.

In sensitivity analysis including patients exempted from referral to pulmonary rehabilitation in the denominator; relative to patients in Welsh practices, patients in English and Scottish practices were still much less likely to receive a pulmonary rehabilitation referral, however, the difference for Northern Irish patients was borderline statistically significant (Fig. 3/Table 2).

**Table 2:** Odds ratios and 95% confidence intervals for receipt of a referral to pulmonary rehabilitation, including patients that were exempted from referral to pulmonary rehabilitation, for each UK country relative to Wales in crude and adjusted models.

	Odds Ratio (95% confidence interval)		
	Crude	Age and gender-adjusted	Age, gender, and comorbidities adjusted
**Referral to pulmonary rehabilitation (including excepted patients)**			
Wales	1	1	1
England	0.21 (0.14–0.32)	0.21 (0.14–0.32)	**0.21 (0.14–0.32)**
Scotland	0.24 (0.15–0.38)	0.23 (0.15–0.37)	**0.23 (0.15–0.37)**
Northern Ireland	0.58 (0.29–1.13)	0.55 (0.28–1.09)	0.55 (0.28–1.08)

Bold text inidicate statistically significant values in the final age, gener, and comorbidites adjusted model.

## Discussion

While it was already clear from the COPD primary care audit that there is shortfall in delivering key aspects of COPD care, it appears that there is some variability between the UK nations, with Scottish practices often performing less well, while English and Northern Irish practices perform similarly to Welsh ones. This could be due to the quality of event recording, quality of care given, or a combination of the two, perhaps driven by participation in the Quality and Outcomes Framework (QOF) pay-for-performance scheme, which Scotland stopped participating in 2016^10^. For example, different national priorities, levels of funding/incentivisation, and therefore availability of programmes may explain differences in the proportion of patients being referred to pulmonary rehabilitation.

Recording of post-bronchodilator spirometry was poor in all nations, and this is likely due to GPs using generic rather than specific post-bronchodilator codes to record the results of spirometry^8,11^. However, even with this poor UK-wide recording of post-bronchodilator spirometry, Welsh primary care practices were still significantly better than other UK practices at confirming, or at least coding confirmation of, airway obstruction. Welsh primary care practices were also significantly better at referring patients to pulmonary rehabilitation than practices in other UK nations. The reason for Welsh practices’ greater performance in confirmation of airway obstruction and referral to pulmonary rehabilitation could perhaps be that participation in the primary care audit has led to an increased awareness of the importance of these interventions and how to accurately code them in the patient’s electronic health record. This is after all the second primary care audit that Wales has participated, with the previous being in 2015^12^. Further supporting this possible explanation, greater referral to pulmonary rehabilitation in Wales is largely driven by greater exception reporting, which requires extra coding in the patient record to exclude patients from the denominator of their QOF payment calculations. Of note, spirometry recording was highly correlated within practices and recording of spirometry varied from 0% to 95% at the practice level. This is a substantial variation in the quality of data recording across practices and recording here could perhaps be improved by increasing GP awareness of the most accurate way to code spirometry results in their GP software package. Additionally, accurate coding of lung function is easier with the Vision software than other packages and this could explain why the recording of spirometry was slightly better in our CPRD GOLD cohort, which comprises practices using Vision, than the audit cohort which comprised almost all practices in Wales. Alternatively, the increased recording of spirometry in Welsh practices could be explained by the Welsh government making it a strategic objective in 2016 which has resulted in the provision of all practices with a standard spirometer and certified training to practice nurses for its use.

QOF may also be a factor in explaining our results. Post-bronchodilator spirometry is financially incentivised through the QOF in Wales^13^, England^14^, and Northern Ireland^15^, and had also been in Scotland until the previous year^16^ (Scotland left QOF in 2016^10^ and the audit was in 2017). With no other differences in QOF incentivisation between the countries, the audit could explain why Welsh practices were significantly better at recording post-bronchodilator spirometry. It’s also interesting to note that Wales was the only country that incentivised referral to pulmonary rehabilitation through the QOF^13–16^ and therefore this financial incentivisation combined with the audit could have helped deliver the substantially better pulmonary rehabilitation referrals in Welsh practices than in practices in the other countries. And in the case of influenza immunisation, which was not financially incentivised through the QOF in Wales^13^, unlike in England^14^ and Northern Ireland^15^ (and in Scotland until the previous year^16^), performance was worse in Welsh practices than in the other countries, which further suggests that the QOF is a factor in the performance of each of the countries.

There are likely other factors at play too, such as the ease of completing a specific element of care, or national or practice focuses. If the audit is leading to improvements in just the coding of care, rather than the care itself, this could perhaps explain why the improvements seen in Wales do seem to be for those areas of care where improved coding could lead to the appearance of better results (such as post-bronchodilator spirometry and pulmonary rehabilitation referral), whereas those areas that are unlikely to be affected by coding issues, such as seasonal influenza immunisation did not see improvement in Wales. Differences in the locations used for key components of healthcare may also explain some of the differences between the countries. For example, if tests are undertaken in hospital, it is possible that the data are not input into the GP computer system. Equally outcomes such as influenza vaccination may be undertaken in a number of settings, and it is possible that although it occurs, it does not get coded in the primary care record. This may also be true for smoking cessation services.

Since devolution in 1999, there have been numerous reports into the impact of divergences of health policy on outcomes in the four UK countries. However, one overarching theme in these reports is that comparisons between the countries is difficult due to inconsistent recording of data in each country^17,18^. Analyses of the QOF have found that patients from all countries generally received best practice care, but Scotland and Northern Ireland performed better at delivering evidence-based care than England and Wales^17,19^. However, these studies used data from 2008/09^19^ and 2010/11^17^ so changes in care quality in each nation over the past 10 years could explain contrasting findings in this study, where Scotland generally performed worst.

The major strength of this study is its size; 56,764 patients from 305 GP practices were included. However, this study is not without limitations; it would have been desirable to adjust for socioeconomic status or deprivation as the UK countries have differing levels of deprivation^20^. Each country has its own measure of multiple deprivations, however, these measures are not comparable between nations^20–24^, and CPRD only provides additional linked data for England. A further limitation is that when making assessments of treatment provided using electronic health records, we only see what has been recorded, which may not always reflect reality. It may be that essential details have been recorded in free text or recorded using different codes than would be expected, and therefore levels of care received may be higher than it appears for items of care that are more complex for GPs to code accurately. There is also a risk that the practices included in our study are slightly larger than the average practice in the UK^25^ and therefore our results may only be generalisable to larger practices rather than all UK practices.

England, Scotland, and Northern Ireland had significantly fewer patients with COPD than Wales that received coded documented confirmation of airways obstruction and referrals to pulmonary rehabilitation. It is possible that national audit in Wales has led to improvements in the delivery of, or at the very least, improvements in the recording of care, that are not being seen in the UK countries without national audits. This highlights the importance of audits such as the NACAP primary care audit for improving quality of care and the recording of that care for benchmarking and future improvement.

## Methods

### Dataset/population

Clinical Practice Research Datalink (CPRD) is part of the Department of Health and Social Care and provides pseudonymised data from participating GP practices across the UK^26^. To complete the analysis we used data from the August 2018 cut of CPRD GP Online Data (GOLD), a database of primary care records from practices that use the Vision GP software package^27,28^. All regions of the UK are well represented in CPRD GOLD and the geographic distribution of practices is detailed by Herrett et al.^27^ in their profile of CPRD GOLD. Our study population was a cohort of COPD patients defined identically to the NACAP 2017 primary care audit^8^: using COPD Read v2 codes validated^29^ for use in primary care electronic health records (EHRs).

### Study design

To achieve our aim of comparing COPD primary care between the UK nations, we completed a replication of the NACAP 2017 primary care audit^8^ in all CPRD GOLD practices and compared results for key care outcomes between practices from each of the UK countries to determine if there are significant differences in the quality of COPD care between the other three UK countries and Wales. This meant that our study design was a cross-sectional study of primary care received by patients with COPD up until 31st March 2017.

Patients were excluded from analyses where any data in the patient record did not meet data quality checks, and patients that were neither male nor female were excluded due to small numbers

### Variables

The exposure variable was the country in which the GP practice is located. The 14 outcomes^30^ used in the NACAP audit were limited to 7 key measures of care (highlighted in bold in Supplementary notes, covering key areas of COPD care: diagnosis, assessment, and high-value care) to keep the analysis focused and reduce the possibility of chance findings. The covariates included were age (categorised into 10-year bands), gender, and the 13 comorbidities included in the NACAP primary care audit^8^ (asthma, bronchiectasis, coronary heart disease, diabetes, heart failure, hypertension, lung cancer, stroke, osteoporosis, anxiety, depression, severe mental illness, and painful condition [defined as prescriptions for 4 or more analgesic or anti-epileptic medications in the absence of an epilepsy diagnosis in the past 12 months]). Comorbidity definitions were identical to those used in the audit^30^. However, as the audit data utilised 5-byte Read V2 codes and CPRD GOLD utilises 7-byte Read V2 codes, additional synonym 7-byte codes were included where present. It was not possible to exactly replicate prescription codelists used in the primary care audit because prescriptions in CPRD GOLD are recorded using gemscript codes instead of Read V2 codes. New prescription codelists were generated by searching for all drug and brand names included in the original primary care audit codelists. Codelists for comorbidity and outcome definitions can be found at 10.5281/zenodo.7080399.

### Statistical analysis

Data were first summarised with frequencies and proportions, and means and standard deviations, as appropriate. The proportion of patients receiving each of the 7 key items of COPD care from the national audit were calculated for each UK country. Mixed-effects logistic regression using a random intercept for practice (to account for clustering of patients within practices) was used to explore the association between the country of general practice and each of the seven key elements of COPD care, generating odds ratios and 95% confidence intervals. The logistic regression models were initially adjusted for age and gender, and then further adjusted for the 13 comorbidities.

A sensitivity analysis was undertaken for referral to pulmonary rehabilitation, including any patients in the denominator (rather than excluding as in initial analyses) who had a Read code in their health record indicating that they should be exempted from referral to pulmonary rehabilitation.

All data management and analyses were completed using Stata 16 MP. Bar charts were created using Microsoft Excel and odds ratio plots were generated using coefplot^31^.

### Reporting summary

Further information on research design is available in the Nature Research Reporting Summary linked to this article.

## Supplementary information

Supplementary notes

REPORTING SUMMARY

## Data Availability

This study used existing data from the UK CPRD EHR database, and this data resource is accessible only to researchers with protocols approved by the CPRD’s independent scientific advisory committee; therefore, no additional unpublished data are available. The study protocol and analysis plan are available upon request to the corresponding author. CPRD GOLD data are available on request from the CPRD. Their provision requires the purchase of a license, and our license does not permit us to make them publicly available. Licences are available from the CPRD (http://www.cprd.com): The Clinical Practice Research Datalink Group, The Medicines and Healthcare products Regulatory Agency, 10 South Colonnade, Canary Wharf, London E14 4PU. We used data from the August 2018 cut of CPRD GOLD and have clearly specified the data selected in our “Methods” section. To allow identical data to be obtained by others following the purchase of a license, we will provide Stata do files required to build the dataset upon request to the corresponding author. Codelists and analysis Stata do files are available at 10.5281/zenodo.7080399.

## Code availability

All analysis computer code are available at 10.5281/zenodo.7080399. Data management computer code are available upon request to the corresponding author.
