# Supplementary material for: Comparison of COPD primary care in England, Scotland, Wales, and Northern Ireland
Source: NPJ Prim Care Respir Med. 2022 Oct 25;32:46. doi: 10.1038/s41533-022-00305-8 (PMC9592607; doi:10.1038/s41533-022-00305-8)
Supplement: Supplementary file 1 — Supplementary notes [file 41533_2022_305_MOESM1_ESM.pdf]

Supplementary notes

2017 NACAP Primary Care Audit queries

1. **Proportion of patients with a post-bronchodilator FEV_1/FVC ratio < 0.7 (latest ever recorded before the audit date).**
2. **Proportion of patients with a chest X-ray 6 months prior to, or within 6 months of first COPD diagnosis.**
3. **Proportion of patients with an MRC score recorded in the year preceding the audit date.**
4. Proportion of patients with FEV_1 percent-predicted recorded in the year preceding the audit date.
5. **Proportion and status of patients asked about tobacco smoking in the year preceding the audit date.**
6. Proportion of patients with 0, 1, or 2 or more exacerbations in the year preceding the audit date (using both GP recorded codes and validated codes (lower respiratory tract infection, oral corticosteroid, and antibiotic codes)[13]).
7. Proportion of patients with an oxygen saturation level of 92% or less who have had arterial blood gas measurement or referral for home oxygen assessment.
8. Proportion of patients who have been prescribed an inhaler who have had their inhaler technique assessed in the year preceding the audit date.
9. **Proportion of patients who have had the influenza immunisation between 1st August 2016 and 31st March 2017.**

10. **Proportion of patients recorded as a current smoker in the 2 years preceding the audit date who have had a referral to a behavioural change intervention *and* had a stop smoking drug prescribed.**

11. **Proportion of (non-exempted) patients referred to pulmonary rehabilitation in the 3 years preceding the audit date with:**

    a. **an MRC score of 3-5.**

    b. any MRC score.

12. Proportion of patients on each type of inhaled therapy (LAMA, LABA, ICS, and their combinations (e.g., LABA & LAMA, triple therapy, etc.)) in the 6 months preceding the audit date.

13. Proportion of patients screened for, or diagnosed with, depression or anxiety in the 2 years preceding the audit date.

14. Proportion of patients on oxygen therapy in the 6 months preceding the audit date.
